# Coraliomargarita algicola sp. nov., isolated from a marine green alga

**DOI:** 10.1099/ijsem.0.006367

**Published:** 2024-05-08

**Authors:** Jae Kyeong Lee, Dae Gyu Choi, Byeong Jun Choi, Jeong Min Kim, Che Ok Jeon

**Affiliations:** 1 Department of Life Science, Chung-Ang University, Seoul 06974, Republic of Korea

**Keywords:** *Coraliomargarita algicola*, new taxa, marine green alga, Verrucomicrobiota

## Abstract

A Gram-stain-negative, facultative aerobic, catalase- and oxidase-positive, non-motile, non-flagellated, and coccus-shaped bacterium, strain J2-16^T^, isolated from a marine green alga, was characterized taxonomically. Strain J2-16^T^ grew at 20–40 °C (optimum, 30 °C), pH 6.0–10.0 (optimum, pH 7.0), and 1.0–4.0 % (w/v) NaCl (optimum, 3.0 %). Menaquinone-7 was identified as the sole respiratory quinone, and major fatty acids (>5 %) were C_18 : 1_* ω*9*c*, iso-C_14 : 0_, C_14 : 0_, anteiso-C_15 : 0_, C_18 : 0_, C_16 : 0_, and C_17 : 1_* ω*8*c*. The polar lipids of strain J2-16^T^ consisted of phosphatidylglycerol, phosphatidylethanolamine, two unidentified phospholipids, and three unidentified lipids. The genome size of strain J2-16^T^ was 5384 kb with a G+C content of 52.0 mol%. Phylogenetic analyses based on 16S rRNA gene and 120 protein marker sequences revealed that strain J2-16^T^ formed a distinct phyletic lineage within the genus *Coraliomargarita*, closely related to *Coraliomargarita sinensis* WN38^T^ and *Coraliomargarita akajimensis* DSM 45221^T^ with 16S rRNA gene sequence similarities of 95.7 and 94.4 %, respectively. Average nucleotide identity and digital DNA–DNA hybridization values between strain J2-16^T^ and *Coraliomargarita* species were lower than 71.2 and 20.0 %, respectively. The phenotypic, chemotaxonomic, and molecular features support that strain J2-16^T^ represents a novel species of the genus *Coraliomargarita*, for which the name *Coraliomargarita algicola* sp. nov. is proposed. The type strain is J2-16^T^ (=KACC 22590^T^=JCM 35407^T^).

## Introduction

The genus *Coraliomargarita* was originally classified within the family *Puniceicoccaceae* of the phylum '*Verrucomicrobia*' with *Coraliomargarita akajimensis* designated as the type species in 2007 [1]. However, recent taxonomic revisions have led to the reclassification of *Coraliomargarita* members into the new family *Coraliomargaritaceae* [2]. As of March 2024, the genus comprises only three validly published species: *Coraliomargarita sinensis*, *Coraliomargarita akajimensis*, and *Coraliomargarita parva* (https://lpsn.dsmz.de/genus/coraliomargarita). *Coraliomargarita* species have been isolated from marine environments, including seawater [1], mangrove sediment [2], and marine solar saltern [3]. These bacteria are Gram-negative, non-flagellated cocci, containing menaquinone-7 (MK-7) as the major respiratory quinone, C_18 : 1_* ω*9*c*, iso-C_14 : 0_, and C_14 : 0_ as the major fatty acids, genomic DNA G+C contents ranging from 53.6 to 56.0 mol%, and phosphatidylglycerol (PG) and phosphatidylethanolamine (PE) as major polar lipids [1,4]. In our investigation of interactions between marine algae and bacteria, we have isolated numerous novel bacteria from the phycosphere of marine algae [5,10]. In this particular study, we isolated a strain designated as J2-16^T^, belonging to the genus *Coraliomargarita*, from a marine green alga. We then analysed its taxonomic characteristics using a polyphasic approach.

## Strain isolation

Strain J2-16^T^ was isolated from the phycosphere of a marine green alga, *Ulva lactuca*, collected from Jajakdo beach (38° 18′ 30″ N 128° 32′ 34″ E) in Gangwon-do, Republic of Korea, as described previously [8]. Initially, the collected green alga was washed using artificial seawater (ASW; 20.0 g NaCl, 2.9 g MgSO_4_, 4.5 g MgCl_2_·6H_2_O, 0.6 g KCl, 1.8 g CaCl_2_·2H_2_O per litre) via vortexing. Subsequently, the washed alga was mechanically homogenized using an Ultra-Turrax homogenizer (IKA) for 10 s, followed by serial dilution in ASW. Aliquots of 100 µl from each serial dilution were plated on marine agar (MA; MBcell) and incubated aerobically at 25 °C for 7 days. Colonies grown on MA were subjected to PCR amplification of their 16S rRNA gene using universal primers F1 (5′-AGAGTTTGATCMTGGCTCAG-3′) and R13 (5′-TACGGYTACCTTGTTACGACTT-3′) [8]. The resulting PCR amplicons were double-digested with restriction enzymes *Hae*III and *Hha*I, and their restriction fragment patterns were analysed on a 2 % agarose gel. Representative amplicons with distinct fragment patterns were sequenced using the universal 340F (5′-CCTACGGGAGGCAGCAG-3′) primer [8] at Macrogen (Seoul, Republic of Korea). The obtained 16S rRNA gene sequences were compared with the sequences of all validly published type species available in the EzBioCloud server (www.ezbiocloud.net/identify) [11]. Based on the results, strain J2-16^T^ was selected for further taxonomic characterizations as a putative novel species of the genus *Coraliomargarita*. Strain J2-16^T^ was routinely cultivated aerobically on MA at 30 °C for 3 days and stored at −80 °C in marine broth (MB; MBcell) containing 15 % (v/v) glycerol. For comparison of phenotypic properties and fatty acid compositions, the type strains *C. sinensis* KCTC 62602^T^ and *C. akajimensis* KCTC 12865^T^ were obtained from their culture collection centres and used as reference strains.

## 16S rRNA gene phylogeny

The 16S rRNA gene amplicon of strain J2-16^T^, generated by F1 and R13 primers, was subsequently sequenced using universal 16S rRNA gene primers 518R (5′-ATTACCGCGGCTGCTGG-3′) and 805F (5′-GATTAGATACCCTGGTAGTC-3′) [7]. Through assembly of sequences obtained from 340F, 518R, and 805F primer-based sequencing, a nearly complete 16S rRNA gene sequence of strain J2-16^T^ (1478 nucleotides) was obtained. Alignment of the 16S rRNA gene sequences of strain J2-16^T^ and closely related valid type strains was conducted using the fast secondary-structure-aware infernal aligner [12]. Phylogenetic trees, incorporating bootstrap values (1000 replications), were reconstructed using the neighbour-joining (NJ), maximum-likelihood (ML), and maximum-parsimony (MP) algorithms with the Kimura two-parameter model, the nearest-neighbour-interchange heuristic search method, and pairwise deletion options, respectively, implemented in the mega11 software [13].

**Fig. 1.**
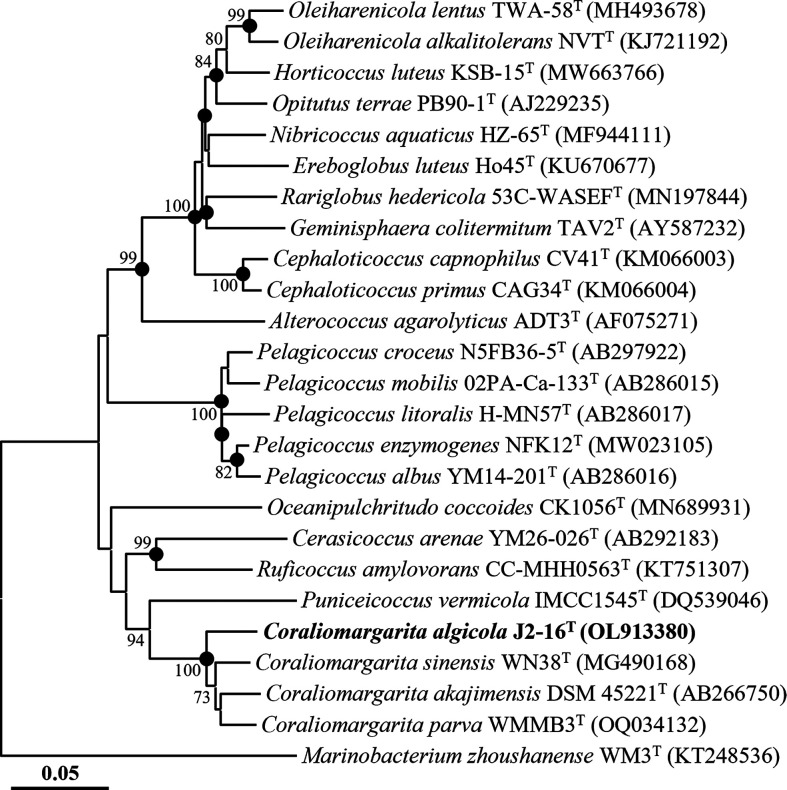
Neighbour-joining tree showing the phylogenetic relationships between strain J2-16^T^ and closely related taxa, based on 16S rRNA gene sequences. Bootstrap values above 70 % are shown on nodes in percentages of 1000 replicates. Filled circles (●) indicate the corresponding nodes that were also recovered in the trees generated with the maximum-likelihood and maximum-parsimony algorithms. *Marinobacterium zhoushanense* WM3^T^ (KT248536) was employed as the outgroup. Bar, 0.05 changes per nucleotide position.

The comparative analysis of 16S rRNA gene sequences revealed that strain J2-16^T^ exhibited the closest relationships with * C. sinensis* WN38^T^ and *C. akajimensis* DSM 45221^T^, with 16S rRNA gene sequence similarities of 95.7 and 94.4 %, respectively. These values fell below the species differentiation threshold of 98.7 % [14], indicating that strain J2-16^T^ likely represents a novel species. Phylogenetic analysis based on 16S rRNA gene sequences using the NJ algorithm demonstrated that strain J2-16^T^ formed a distinct phyletic lineage with members of the genus *Coraliomargarita*, supported by a bootstrap value of 100 % (Fig. 1). Phylogenetic trees reconstructed using the ML and MP algorithms also supported the placement of strain J2-16^T^ within the genus *Coraliomargarita* (Fig. S1, available in the online version of this article). These combined comparative and phylogenetic analyses based on 16S rRNA gene sequences suggest that strain J2-16^T^ may be classified as a novel species within the genus *Coraliomargarita*.

## Genome features

The genomic DNA of strain J2-16^T^ was extracted from cells cultured in MB using the Wizard Genomic DNA purification kit (Promega) following the manufacturer’s instructions, and sequenced using an Oxford Nanopore MinION sequencer (ONT). The resulting sequencing reads of strain J2-16^T^ were *de novo*-assembled using Unicycler (version 0.4.7) [15]. The quality of the assembled genome was assessed for completeness and contamination rates using CheckM2 software (version 1.0.2) [16]. The complete genome sequence of strain J2-16^T^ was deposited in GenBank under the accession number CP138858 and annotated using the NCBI Prokaryotic Genome Annotation Pipeline [17]. A phylogenetic analysis was conducted using the genomes of strain J2-16^T^ and closely related type strains. The amino acid sequences of 120 bacterial marker proteins were extracted from these genomes, concatenated, and aligned using the Genome Taxonomy Database Toolkit (GTDB-Tk) [18]. A phylogenomic tree, incorporating bootstrap values (1000 replications), was reconstructed based on the ML algorithm using mega11 software. Average nucleotide identity (ANI) and digital DNA–DNA hybridization (dDDH) values among the genomes of strain J2-16^T^, *C. sinensis* WN38^T^, and *C. akajimensis* DSM 45221^T^ were calculated using the Orthologous Average Nucleotide Identity Tool (OAT) software available on the EzBioCloud web server (www.ezbiocloud.net/sw/oat) [19] and the web-based Genome-to-Genome Distance Calculator version 3.0 (https://ggdc.dsmz.de/ggdc.php) [20], respectively.

The assembly of genome sequencing reads from strain J2-16^T^ resulted in a complete single genome approximately 5384 kb in size (with an average genome coverage of 142.0×) without any plasmids. The 16S rRNA gene sequence identified in the genome of strain J2-16^T^ was consistent with that obtained via PCR-based sequencing. Assessment using CheckM2 indicated genome completeness and contamination rates of 94.1 and 0.1 %, respectively, meeting the criteria (≥90 % completeness and ≤10 % contamination) for high-quality genomes [16]. The genome of strain J2-16^T^ harboured a total of 4387 genes, comprising 4306 protein-coding genes, 25 pseudogenes, two rRNA operons containing two copies each of 16S, 5S, and 23S rRNA genes, and 46 tRNA genes. General genomic features of strain J2-16^T^ are summarized and compared with closely related type strains of the genus *Coraliomargarita* in Table 1. The DNA G+C content of strain J2-16^T^, calculated from the whole genome, was 52.0 mol%, slightly lower than that of other *Coraliomargarita* species. These characteristics, including genome size, DNA G+C content, and gene contents, distinctly set strain J2-16^T^ apart from other closely related *Coraliomargarita* species.

**Table 1.** General genomic features and genome relatedness of strain J2-16^T^ and closely related type strains of the genus *Coraliomargarita* Strains: 1, J2-16^T^ (CP138858); 2, *C. sinensis* WN38^T^ (QHJQ00000000); 3, *C. akajimensis* DSM 45221^T^ (CP001998) [5]. na; Not available.

Feature	1*	2*	3†
Genome size (kb)	5384	3570	3751
Genome status	Complete	Draft	Complete
No. of contigs	1	36	1
G+C content (mol%)	52.0	54.5	53.6
No. of total genes	4387	3058	3192
No. of protein-coding genes	4306	2984	3137
No. of pseudogenes	25	26	17
No. of rRNA (5S, 16S, 23S) operons	2	1	2
No. of tRNA genes	46	41	na
No. of total CAZy‡ genes	306	127	194
Glycoside hydrolase	214	68	106
Glycosyltransferase	43	33	51
Carbohydrate-binding module	26	13	30
Carbohydrate esterase	17	9	4
Polysaccharide lyase	6	3	2
Auxiliary activities	0	1	1

*The genomic features were analyzedanalysed using the NCBI pProkaryotic gGenome annotation pipeline (www.ncbi.nlm.nih.gov/genome/annotation_prok/).

**†The genomic characteristics were obtained from Mavromatis *et al*. [5].

†‡The carbohydrate active enzyme (CAZy) genes were identified using the dbCAN3 meta server (https://bcb.unl.edu/dbCAN2/blast.php) [22].

**Fig. 2.**
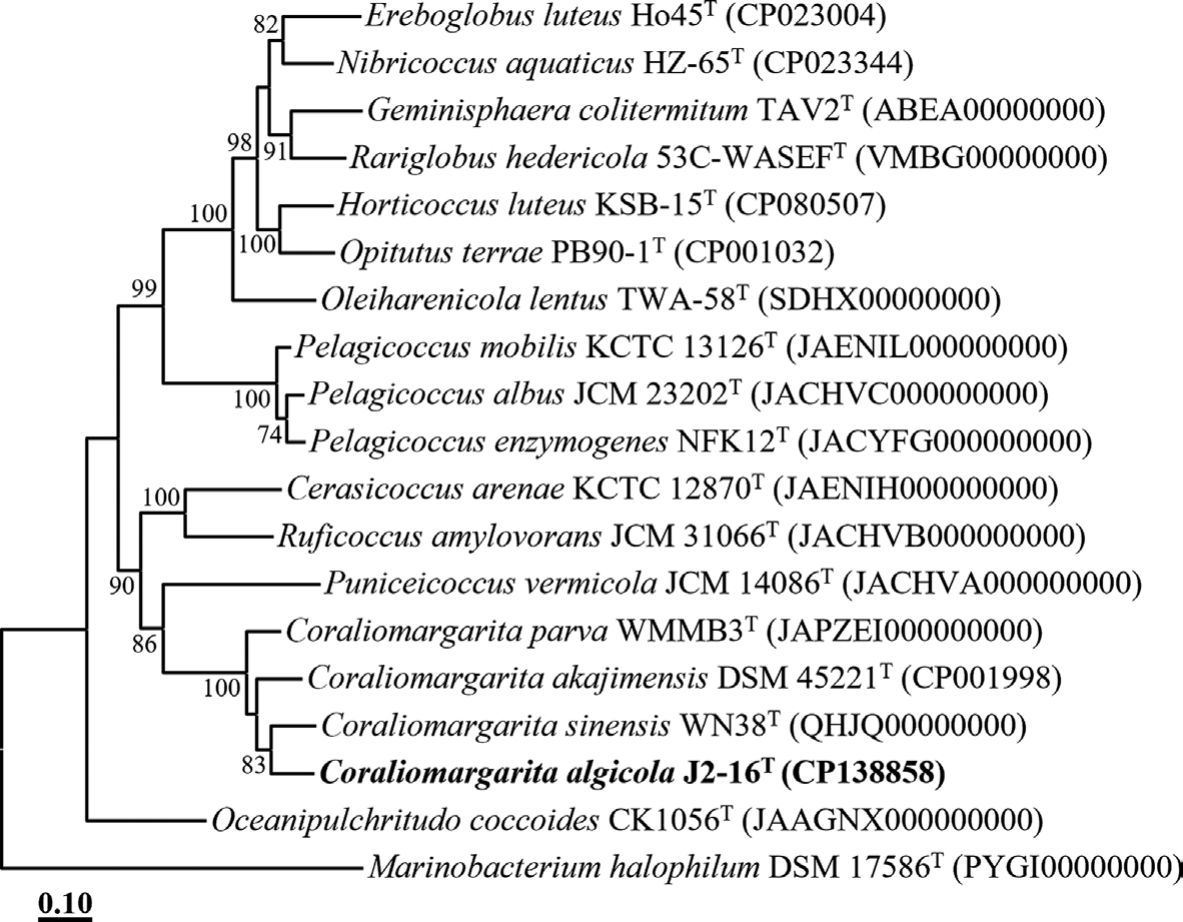
Maximum-likelihood tree showing the phylogenetic relationships between strain J2-16^T^ and closely related taxa, based on concatenated amino acid sequences of 120 bacterial marker proteins. Bootstrap values above 70 % are shown on nodes in percentages of 1000 replicates. *Marinobacterium halophilum* DSM 17586^T^ (PYGI00000000) was employed as the outgroup. Bar, 0.10 changes per amino acid position.

The phylogenetic analysis, based on the concatenated protein sequences of 120 ubiquitous single-copy marker genes (bac120 marker set), also indicated that strain J2-16^T^ formed a phyletic lineage with members of the genus *Coraliomargarita* (Fig. 2), consistent with the findings from the 16S rRNA gene sequence-based analyses. The ANI and dDDH values between strain J2-16^T^ and closely related type strains, *C. akajimensis* DSM 45221^T^ and *C. sinensis* WN38^T^, were 71.2 and 20.0 %, and 70.4 and 19.9 %, respectively (Table S1), significantly below the thresholds for prokaryotic species delineation (ANI, ~95 %; dDDH, 70 %) [14]. These results suggest that strain J2-16^T^ represents a distinct species within the genus *Coraliomargarita*. In conclusion, the genome relatedness and phylogenomic analyses of strain J2-16^T^ with closely related *Coraliomargarita* type strains strongly support its classification as a novel species within the genus *Coraliomargarita*.

## Algal symbiosis-associated genes

Bacteria inhabiting marine ecosystems are recognized for their ability to degrade algal polysaccharides and utilize algal cell wall constituents to support their growth [21]. Consequently, we identified carbohydrate active enzymes (CAZy) genes in strain J2-16^T^ and closely related *Coraliomargarita* species using the dbCAN3 meta server (https://bcb.unl.edu/dbCAN2/blast.php) [22]. Our analysis revealed that the genome of strain J2-16^T^ was predicted to contain a total of 306 genes encoding various CAZy enzymes, representing a higher abundance compared to closely related strains, *C. sinensis* WN38^T^ and *C. akajimensis* DSM 45221^T^, which possess 127 and 194 CAZy genes, respectively (Table 1). The CAZy genes of strain J2-16^T^ were classified into five distinct classes: 214 glycoside hydrolase (GH), 43 glycosyltransferase (GT), 26 carbohydrate-binding module (CBM), and 17 carbohydrate esterase (CE), with the absence of the auxiliary activities (AA) class. The higher abundance of these CAZy category genes compared to other reference *Coraliomargarita* strains (Table 1) indicates that strain J2-16^T^, isolated from the phycosphere of a marine green alga, likely possesses a more versatile capability to metabolize cell-wall components of algae compared to other *Coraliomargarita* species.

## Morphology and physiology

Growth of strain J2-16^T^ was evaluated at 30 °C for 3 days on various bacteriological agar media, including MA, Reasoner's 2A (R2A) agar (MBcell), Luria–Bertani (LB) agar (MBcell), tryptic soy agar (MBcell), and nutrient agar (MBcell), supplemented with NaCl to achieve final concentrations of approximately 2 % (w/v). Additionally, the growth of strain J2-16^T^ was examined at different temperatures (ranging from 5 to 45 °C in 5 °C intervals) on MA for 3 days. Evaluation of growth at various pH values (ranging from pH 4.0 to 11.0 in 1.0 pH unit intervals) was conducted in MB at 30 °C for 3 days. MB media with desired pH levels were prepared using sodium citrate (pH 4.0–5.0), sodium phosphate (pH 6.0–8.0), and sodium carbonate–bicarbonate (pH 9.0–11.0) buffers [23], with pH adjustment after sterilization (121 °C for 15 min). Growth in different NaCl concentrations (ranging from 0 to 10 % in 1.0 % intervals) was assessed using MB medium prepared in the laboratory according to its composition. Anaerobic growth of strain J2-16^T^ was evaluated by streaking it on MA and monitoring growth after 21 days of incubation at 30 °C under anaerobic conditions facilitated by the GasPak Plus system (BBL).

Cell morphology and the presence of flagella were examined using a transmission electron microscope (JEM-1010, jeol) and a phase-contrast microscope (Carl Zeiss Axio Scope.A1), respectively, with cells grown on MA at 30 °C for 3 days. Gram staining was performed using a Gram stain kit (bioMérieux) following the manufacturer’s instructions. Catalase and oxidase activities of strain J2-16^T^ were determined by observing the production of oxygen bubbles in a 3 % (v/v) aqueous hydrogen peroxide solution and the oxidation of 1 % (w/v) tetramethyl-*p*-phenylenediamine (Merck), respectively [24,25]. The following properties of strain J2-16^T^ and two *Coraliomargarita* reference strains were concurrently assessed under the same conditions at 30 °C. Hydrolysis of Tween 20, Tween 80, casein, starch, aesculin, and tyrosine was evaluated on MA according to previously described methods [24,25]. Further biochemical features were examined using the API 20NE kit from bioMérieux, following the manufacturer’s instructions, except that cells resuspended in ASW were used as an inoculum.

Strain J2-16^T^ demonstrated robust growth on MA, with relatively favourable growth observed on R2A agar containing 2.0 % NaCl; however, it did not grow on nutrient agar, LB agar, or tryptic soy agar containing 2.0 % NaCl. Cells of strain J2-16^T^ were Gram-stain-negative and non-motile cocci, measuring approximately 0.7–0.8 µm in diameter (Fig. S2). Anaerobic growth was observed after 21 days of incubation on MA at 30 °C, unlike other *Coraliomargarita* reference strains. Phenotypic properties of strain J2-16^T^, including flagellar motility, nitrate reduction, hydrolysis of tyrosine, gelatin, Tween 20, Tween 80, and casein, activity of arginine dihydrolase and urease, assimilation of d-glucose, l-arabinose, d-mannose, potassium gluconate, capric acid, trisodium citrate, and phenylacetic acid, and indole production, were common with other reference strains. However, other phenotypic properties, such as anaerobic growth, hydrolysis of starch and aesculin, activity of catalase, oxidase, and *β*-glucosidase, and assimilation of d-mannitol, maltose, *N*-acetyl-glucosamine, adipic acid, and malic acid, differentiated strain J2-16^T^ from closely related type strains of the genus *Coraliomargarita* (Table 2).

**Table 2.** Comparisons of phenotype characteristics of strain J2-16^T^ and closely related type strains of the genus *Coraliomargarita* Strains: 1, J2-16^T^ (this study); 2, *C. sinensis* KCTC 62602^T^ [4]; 3, *C. akajimensis* KCTC 12865^T^ [1]. All strains are positive for the activity* of *β*-galactosidase. All strains are negative for the following characteristics: flagella motility, nitrate reduction*, hydrolysis* of tyrosine, gelatin, Tween 20, Tween 80, and casein, activity* of arginine dihydrolase and urease, assimilation* of d-glucose, l-arabinose, d-mannose, potassium gluconate, capric acid, trisodium citrate, and phenylacetic acid, indole production*, and glucose fermentation*. +, Positive; −, negative; w, weakly positive; na, not available.

Characteristic	1	2	3
Source	Marine alga	Marine solar saltern	Seawater
Colony colour	White	White	White
O_2_ requirement	Facultatively aerobic	Obligately aerobic	Obligately aerobic
Catalase	w	+	–
Oxidase	+	–	+
Growth range of:			
Temperature (°C)	20–40	15–40	na
NaCl (%)	1–4	1–10	1–5
pH	6–10	6–9	7–9
Hydrolysis* of:			
Starch	+	–	–
Aesculin	–	+	+
Enzyme activity* of:			
*β*-Glucosidase	–	+	+
Assimilation* of			
d-Mannitol, maltose	+	–	–
*N*-Acetyl-glucosamine	–	+	–
Adipic acid, malic acid	–	–	+

*These analyses were conducted under the same conditions in this study.

## Chemotaxonomic characteristics

Isoprenoid quinones of strain J2-16^T^ were extracted from cells cultured in MB at 30 °C until reaching the exponential growth phase, following the procedure outlined by Minnikin *et al*. [26]. The quinones were analysed using an HPLC system (LC-20A; Shimadzu) equipped with a reversed-phase Kromasil column (250×4.6 mm; Akzo Nobel Center) and an SPD-M20A diode array detector (Shimadzu). Methanol–isopropanol (2 : 1, v/v) served as the eluent, and the flow rate was set at 1 ml min^−1^. Strain J2-16^T^ and two *Coraliomargarita* type strains were cultivated in MB at 30 °C and harvested at the same growth phase (exponential growth phase, optical density, OD_600_=0.8) for cellular fatty acid analysis. Cellular fatty acids were saponified, methylated, and extracted according to the standard midi protocol. Fatty acid methyl esters were analysed using gas chromatography (Hewlett Packard 6890) and identified using the RTSBA6 database of the Microbial Identification System (Sherlock version 6.0B) [27]. Polar lipids of strain J2-16^T^ were extracted from cells harvested during their exponential growth phase and analysed by two-dimensional TLC, following the procedure described by Minnikin *et al*. [28]. Different reagents were used to detect various polar lipids: 10 % ethanolic molybdophosphoric acid reagent (for total polar lipids), ninhydrin (for aminolipids), Dittmer–Lester reagent (for phospholipids), and α-naphthol–sulphuric acid (for glycolipids). PG and PE identified from the polar lipid analysis were confirmed using standard polar lipid compounds purchased from Sigma-Aldrich.

MK-7 was identified as the sole respiratory quinone in strain J2-16^T^, consistent with findings in all other members of the genus *Coraliomargarita* [1,4]. The major cellular fatty acids (>5 % of the total fatty acids) included C_18 : 1_* ω*9*c*, iso-C_14 : 0_, C_14 : 0_, anteiso-C_15 : 0_, C_18 : 0_ C_16 : 0_, and C_17 : 1_* ω*8*c*. While the overall fatty acid profiles of strain J2-16^T^ were similar to those of other reference *Coraliomargarita* species, there were some differences in the respective proportions of certain fatty acids, such as C_16 : 0_, C_17 : 1_* ω*8*c*, anteiso-C_17 : 0_, and C_16 : 0_ 3-OH (Table S2). Major polar lipids detected in strain J2-16^T^ included PG, PE, two unidentified phospholipids, and three unidentified lipids (Fig. S3), with a profile resembling those of other closely related *Coraliomargarita* species [4].

## Taxonomic conclusion

In conclusion, the phylogenetic, genomic relatedness, phenotypic, physiological, biochemical, and chemotaxonomic properties support the identification of strain J2-16^T^ as representing a novel species of the genus *Coraliomargarita*, for which the name *Coraliomargarita algicola* sp. nov. is proposed.

## Description of *Coraliomargarita algicola* sp. nov.

*Coraliomargarita algicola* [al.gi′co.la. L. fem. n. *alga*, a seaweed; L. suffix. -*cola*, (from L. masc. or fem. n. *incola*) inhabitant, dweller; N.L. fem. n. *algicola*, an alga dweller].

Colonies on MA are circular, convex, smooth, and white in colour. Cells are Gram-stain-negative, facultative aerobic, non-motile, and non-flagellated cocci. Growth occurs at 20–40 °C (optimum, 30 °C), pH 6.0–10.0 (optimum, pH 7.0), and 1.0–4.0 % (w/v) NaCl (optimum, 3.0 %). Oxidase- and catalase-positive. Starch is hydrolysed, but Tween 20 and Tween 80, aesculin, tyrosine, casein, and gelatin are not. Nitrate is not reduced to nitrite. Indole production and d-glucose fermentation are negative. *β*-Galactosidase activity is positive, but arginine dihydrolase, urease, and *β*-glucosidase activities are negative. Assimilation of d-mannitol and maltose is positive, but assimilation of d-glucose, l-arabinose, d-mannose, potassium gluconate, *N*-acetyl-glucosamine, capric acid, adipic acid, malic acid, trisodium citrate, and phenylacetic acid is negative. MK-7 is the sole respiratory quinone. PG, PE, two unidentified phospholipids, and three unidentified lipids are detected as major polar lipids. Major cellular fatty acids (>5 %) are C_18 : 1_* ω*9*c*, iso-C_14 : 0_, C_14 : 0_, anteiso-C_15 : 0_, C_18 : 0_, C_16 : 0_, and C_17 : 1_* ω*8*c*.

The type strain is J2-16^T^ (=KACC 22590^T^=JCM 35407^T^), isolated from the phycosphere of a marine green alga, *Ulva lactuca*, collected in the Republic of Korea. The genome size and DNA G+C content of the type strain are 5384 kb and 52.0 mol% (calculated from the whole genome sequence), respectively. The GenBank accession numbers of the 16S rRNA gene and genome sequences of strain J2-16^T^ are OL913380 and CP138858, respectively.

## supplementary material

10.1099/ijsem.0.006367 Uncited Supplementary Material 1.

## Abbreviations

ANI: average nucleotide identity
ASW: artificial seawater
CAZy: carbohydrate active enzymes
dDDH: digital DNA–DNA hybridization
MA: marine agar
MB: marine broth
MK-7: menaquinone-7
ML: maximum-likelihood
MP: maximum-parsimony
NJ: neighbour-joining
PE: phosphatidylethanolamine
PG: phosphatidylglycerol
R2A: Reasoner's 2A
