# Supplementary material for: Coraliomargarita algicola sp. nov., isolated from a marine green alga
Source: Int J Syst Evol Microbiol. 2024 May 8;74(5):006367. doi: 10.1099/ijsem.0.006367 (PMC11165907; doi:10.1099/ijsem.0.006367)
Supplement: Uncited Supplementary Material 1. [file ijsem-74-06367-s001.pdf]

Supplementary Information

Fig. S1. Maximum-likelihood (A) and maximum-parsimony (B) trees showing the phylogenetic relationships between strain J2-16^{T} and their closely related taxa, based on 16S rRNA gene sequences. Bootstrap values above 70% are shown on nodes in percentages of 1000 replicates. *Marinobacterium zhoushanense* WM3^{T} (KT248536) was employed as the outgroup. Scale bars in panels A and B represent substitutions per nucleotide and over the entire sequences, respectively.

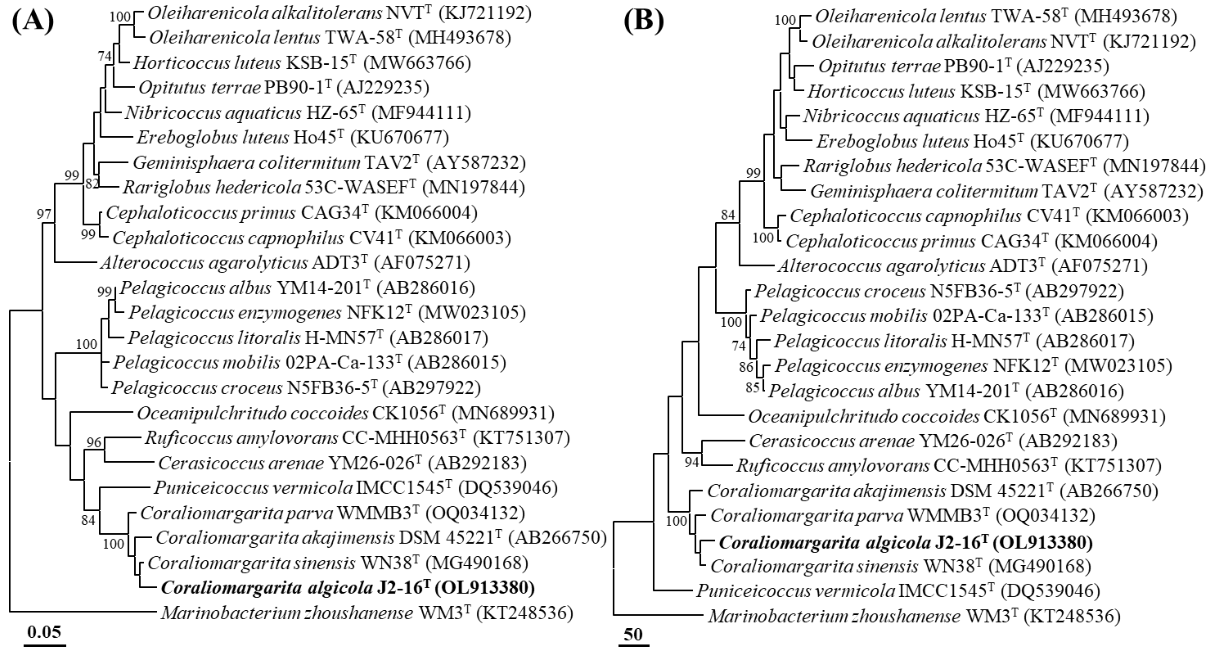

Fig. S2. A transmission electron micrograph of negatively stained cells using 2% uranyl acetate showing the general cellular morphology of strain J2-16^{T} grown on marine agar at 30°C for 3 days. Bar, 1 µm.

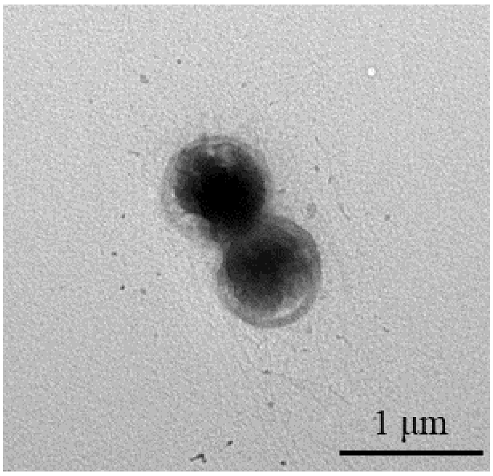

Fig. S3. Two-dimensional thin-layer chromatograms (TLC) showing the polar lipid profiles of strain J2-16^T. Solvent systems: (I) chloroform-methanol-water (65:25:4, v/v/v) and (II) chloroform-acetic acid-methanol-water (80:15:12:4, v/v/v/v). The TLC plates were sprayed with 10% ethanolic molybdophosphoric acid (A), ninhydrin (B), Dittmer-Lester (C), and *α*-naphthol/sulfuric acid (D) for the detection of total polar lipids, aminolipids, phospholipids, and glycolipids, respectively. PG, phosphatidylglycerol; PE, phosphatidylethanolamine; PL, unidentified phospholipid; L, unidentified lipid.

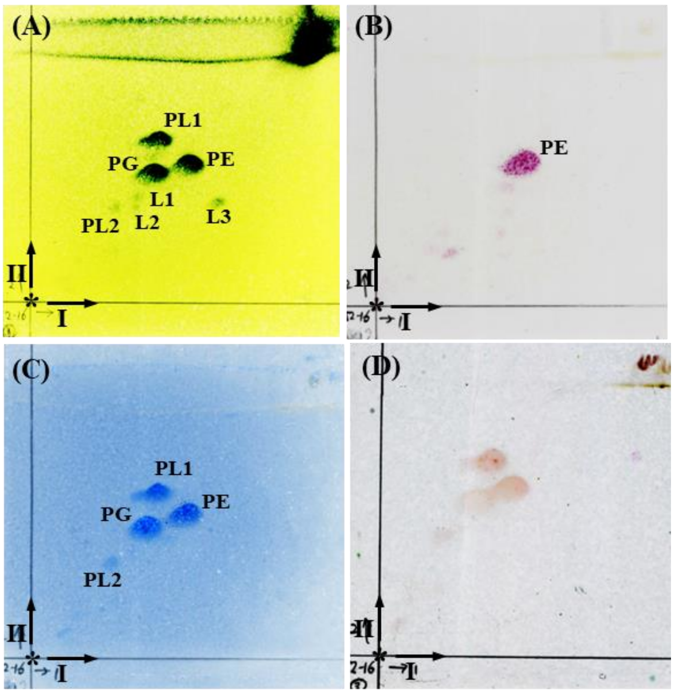

Table S1. Genome relatedness among strain J2-16^T and closely related type strains of the genus *Coraliomargarita*

Taxa: 1, strain J2-16^T (CP138858); 2, *C. sinensis* WN38^T (QHJQ00000000); 3, *C. akajimensis* DSM 45221^T (CP001998).

		dDDH† value (%)		
		**1**	**2**	**3**
ANI† value (%)	**1**	–	20.0	19.9
	**2**	71.2	–	20.6
	**3**	70.4	70.5	–

†ANI, average nucleotide identity; dDDH, digital DNA-DNA hybridization.

Table S2. Comparison of the cellular fatty acid compositions (%) between strain J2-16^T and closely related type strains of the genus *Coraliomargarita*

Taxa: 1, strain J2-16^T; 2, *C. sinensis* KCTC 62602^T; 3, *C. akajimensis* KCTC 12865^T. All data were obtained from this study. Data are expressed as percentages for the total fatty acids, and fatty acids less than 1.0% in all strains are not indicated. Major components (>5.0%) are highlighted in bold. Symbols: TR, trace amount (<1.0%); –, not detected.

**Fatty acid**	**1**	**2**	**3**
Saturated:			
$C_{12:0}$	TR	1.1	TR
$C_{14:0}$	**9.1**	**5.8**	**11.4**
$C_{16:0}$	**5.7**	3.3	4.0
$C_{17:0}$	2.7	1.2	TR
$C_{18:0}$	**7.4**	**6.2**	**7.3**
$C_{19:0}$	1.4	3.1	1.0
$C_{20:0}$	TR	2.4	4.7
Unsaturated:			
$C_{17:1}$ $\omega 8c$	**5.5**	2.7	TR
$C_{18:1}$ $\omega 9c$	**25.4**	**16.5**	**28.7**
$C_{20:1}$ $\omega 9c$	TR	TR	4.2
Branched:			
iso-$C_{14:0}$	**17.4**	**12.4**	**12.6**
anteiso-$C_{15:0}$	**7.6**	**14.1**	**5.8**
iso-$C_{16:0}$	2.7	3.5	4.7
anteiso-$C_{17:0}$	1.0	**6.4**	1.0
iso-$C_{18:0}$	3.8	**8.0**	2.1
iso-$C_{19:0}$	TR	1.5	TR
anteiso-$C_{19:0}$	TR	2.0	TR
iso-$C_{20:0}$	–	1.3	TR
Hydroxy:			
$C_{12:0}$ 3-OH	TR	TR	4.0
$C_{14:0}$ iso 3-OH	1.7	2.6	TR
$C_{16:0}$ 3-OH	–	–	1.6
Summed feature*:			
6	1.3	1.6	TR

*Summed features are fatty acids that cannot be resolved reliably from another fatty acid using the chromatographic conditions chosen. The MIDI system groups these fatty acids together as one feature with a single percentage of the total. Summed feature 6, $C_{19:1}$ $\omega 11c$ and/or $C_{19:1}$ $\omega 9c$.
